# Exposure to *Toxocara* spp. and *Ascaris lumbricoides* infections and risk of allergic rhinitis in children

**DOI:** 10.1186/s13223-020-00468-4

**Published:** 2020-07-30

**Authors:** Iraj Mohammadzadeh, Sorena Darvish, Seyed Mohammad Riahi, Solmaz Alizadeh Moghaddam, Mohammad Pournasrollah, Mousa Mohammadnia-Afrozi, Ali Rostami

**Affiliations:** 1 Non-Communicable Pediatric Diseases Research Center, Health Research Institute, Babol University of Medical Sciences, Babol, Iran; 2 Infectious Diseases and Tropical Medicine Research Center, Health Research Institute, Babol University of Medical Sciences, Babol, Iran; 3 Department of Epidemiology and Biostatistics, Faculty of Health, Cardiovascular Diseases Research Center, Birjand University of Medical Sciences, Birjand, Iran; 4 Immunoregulation Research Center, Health Research Institute, Babol University of Medical Sciences, Babol, Iran

**Keywords:** *Toxocara* spp., *Ascaris lumbricoides*, Seropositivity, ELISA, Allergic rhinitis

## Abstract

**Background:**

Substantial experimental studies suggest a role for helminthes infections in the pathogenesis of allergies, but epidemiologic data have been inconsistent. Unlike to asthma, the association between helminthes infection and allergic rhinitis (AR) has been poorly studied. Therefore, we sought to evaluate the association between exposure to *Ascaris* and *Toxocara* infections and AR.

**Methods:**

We did an age- and gender-matched case–control study of 81 children with physician-confirmed AR and 101 control subjects in a referral hospital for pediatric diseases in northern Iran. Exposure to *Ascaris* and *Toxocara* infections was evaluated by anti-*A. lumbricoides*- and anti-*Toxocara*- IgG antibodies using a commercial enzyme-linked immunosorbent assay. Associations were determined using multivariate logistic regression.

**Results:**

*Ascaris* seropositivity was higher in children with rhinitis than in controls (12.34 vs. 3.96%). *Ascaris* seropositivity was positively associated with AR in univariate analysis (OR, 3.42; 95% CI 1.03–11.3; P value = 0.035), but this association was not significant after adjustment for potential confounders (OR, 1.85; 95% CI 0.42–8.18). Also *Toxocara* seropositivity was higher in children with AR than in healthy subjects (3.7% vs. 0.99), indicating non-significant association with AR in both univariate (OR, 3.84; 95% CI 0.39–37.7) and multivariate analyses (OR, 0.8; 95% CI 0.04–15.44).

**Conclusion:**

Our results revealed that AR is not associated with seropositivity to *Ascaris* and *Toxocara* infections in general; however, a higher seropositivity rate was found for both parasites in children with AR. More studies with longitudinal design and larger sample size are needed to elucidate this association.

## Background

Allergic rhinitis (AR) is one of the most prevalent and increasing inflammatory allergic disorders, affecting about 40% of the world population in all ages, with a great peak in the childhood [1, 2]. It is a global health problem, and although isn’t life-threatening but can cause significant adverse impacts on quality of life and emotional well-being, including poor sleep quality, poor performance in work or school, impaired cognitive function, poor social life, fatigue, and depression and anxiety [3]. AR is caused by IgE-mediated early- and late-phase hypersensitivity responses and characterized by pruritus, sneezing, nasal obstruction and blockage, nasal itching and rhinorrhea [4]. AR is strongly linked to other atopic respiratory diseases, such as asthma, and therefore have common environmental and genetic origin [5]. Studies indicated that AR is associated with many genetic loci on chromosomes 2, 5, 6, 7, 11, 13, 16, and 20 [6]. Other predictors to development of AR in children are including environmental pollution, birth during a pollen season, high socioeconomic status, ethnic origin, heavy maternal smoking during the first year of life, exposure to indoor allergens such as animal dander and dust mites, high concentrations in serum of IgE (> 100 IU/mL before age 6 years), positive allergen skin prick tests and early introduction of foods or formula [1, 7]. Moreover although some epidemiologic studies have shown that environmental exposure to products (e.g., endotoxins and lipopolysaccharides) of infectious agents such as *Mycobacterium* spp, hepatitis A, and *Toxoplasma gondii*, have a protective effect against development of AR, but infection with soil transmitted helminthes (such as *Enterobius vermicularis*, *Toxocara* spp. and *Ascaris lumbricoides*) have yielded different results and most of epidemiologic studies and meta-analyses indicated that these infections are risk factors for development of allergic disorders.

Both *Toxocara* spp. and *A. lumbricoides* are ascarid nematodes with worldwide distribution [8, 9]. It is estimated that, around the world, about 1.4 billion people are seropositive for *Toxocara* infection [10–12] and 700 million people are infected with *Ascaris* infection [9, 13]. These parasites have a fecal–oral transmission and poor sanitation status, contact with soil and animals, drinking untreated water, eating unwashed vegetables and crowded or high-density living conditions, as found in tropical developing countries are major risk factors of these infections [10, 13]. Elevated levels of serum IgE and eosinophilia are the common indicators for both allergic disorders and helminthic infections, therefore it is hypothesized that intestinal or tissue helminthic infections may also play an etiological role in development of allergic disorders [9, 14–17]. Two comprehensive meta-analyses have showed that *Toxocara* spp. and *A. lumbricoides* infections have a positive association to development of asthma [14, 18], but studies evaluating the association between these infection and allergic rhinitis are very rare. To our knowledge there are four studies evaluating association between *Toxocara* infection and AR [17, 19–21], showing conflict results, although we have found only two relevant studies with respect to *A. lumbricoides* infection [21, 22].

Northern Iran is an endemic area for many parasitic infections including *Toxocara* and *A. lumbricoides* infection [9, 23–25]. Also prevalence of AR is high in this area [26, 27]. Therefore the main objective of this study was to evaluate the association between *Toxocara* and *A. lumbricoides* infection and development of AR in children in this area.

## Materials and methods

### Study design and population

In this matched case–control study, cases were 81 children (aged 2–15 years) with physician-confirmed AR diagnosed according to Allergic Rhinitis and Its Impact on Asthma (ARIA) guidelines [28] at the Amirkola Hospital in Mazandaran province, Iran, which is largest referral hospital for pediatric disease in northern Iran. Following clinical symptoms related with AR were assessed in all patients: sneezing, rhinorrhea, nasal obstruction, itching, and facial features (shiners, allergic salute, mouth breathing, nasal crease, infraorbital fold, and conjunctivitis). Controls were 101 age- and sex- matched healthy children with no history of atopy or asthma and other allergic disorders and no current gastrointestinal disorders. Children were excluded if they had other relevant diseases such as respiratory tract infections, sinusitis or asthma; had used corticosteroids and inhaled corticosteroids within the last month; had used antiparasitic drugs or immunotherapy in the 6 months prior to the study; and had any other known medical condition such as hepatosplenomegaly, generalized lymphadenopathy, or ocular symptom. The ethics review board of Babol University of Medical Sciences approved the study protocol (IR.MUBABOL.HRI.REC.1397.289 and IR.MUBABOL.HRI.REC.1397.288). The parents or legal guardians of all recruited children in the study signed an informed consent.

### Sample collection and serological assays

Blood samples (3–5-mL) were taken from all cases and controls with the use of vacutainer tubes and sera were separated after centrifugation at 1000*g* for 5 min. Serum samples were collected and stored at − 20 °C. Exposure to *Ascaris* and *Toxocara* infections was evaluated by anti-*A. lumbricoides*- and anti-*Toxocara*- IgG antibodies using a commercial enzyme linked immunosorbent assay (ELISA) kit (NovaTec Immunodiagnostics, Dietzenbach, Germany) following the manufacturer’s instructions. The sensitivity and specificity for this kits have been reported to be > 95%. According to the manufacturer’s recommendation, results were reported in International Units (IU). Sera with values of < 9.0, 9.0–11.0, and > 11.0 IU/mL were considered negative, suspect (gray zone), and positive, for antibodies to toxocariasis and ascariasis. Moreover total IgE and, absolute eosinophilic count (AEC) were determined for case group.

### Statistical analysis

All statistical analyses were done by SPSS Statistics software, version 21 (IBM, Armonk, NY, USA). Descriptive data for cases and controls were summarized using the relative frequency with an exact binomial 95% confidence interval (CI). Chi square test and crude odds ratio with 95% confidence interval were used to determine the relationship between demographic variables and AR. The effects of *Toxocara* and *Ascaris* infections on the risk of developing AR were expressed by logistic regression analysis. In this study, univariate logistic regression was performed to determine the factors affecting the AR, then variables with *P* value below 0.1 were included in the multivariate logistic regression. Due to the importance of two variables, family atopic history and parent’s smoking, the modeling was done in three models, in which model 1 included age, sex, residence, parents education, mother’s occupation; model 2 included model 1 and family atopic history; model 3 included model 2 and parents smoking. The logistic regression models was evaluated by Hosmer–Lemeshow test and the receiver operating characteristic (ROC) curves analysis and the area under the curve (AUC) [29]. The AUC value is between zero and 100. The model with higher AUC is considered to be the optimal model. A *P*-value of less than 0.05 was accepted as statistically significant.

## Results

There were 81 children with rhinitis and 101 matched controls. The median age of the cases and controls was 7.04 ± 2.69 and 6.52 ± 3.57 years, respectively. There were 44 (54.3%) boys in the case- and 57 (56.4%) boys in control- groups. The proportions of rural children of the case and control groups 71.6% and 51.5%, respectively. Thirty-five (43.2%) of children had family atopic history. More demographic features for both cases and controls are shown in Table 1. Univariate analysis revealed that children with rhinitis were more likely to have parents with high levels of education (OR, 4.53; 95% CI 2.37–8.68; *P* value < 0.001), working mothers (OR, 4.70; 95% CI 1.88–11.73; *P* value < 0.001), family atopic history (OR, 14.6; 95% CI 5.37–39.7; *P* value < 0.001) and smoker parents (OR, 3.52; 95% CI 1.65–7.5; *P* value < 0.001) (Table 1).

**Table 1:** Demographic characteristics and relative frequency of Ascariasis in children with rhinitis and healthy controls

Variable	Children with rhinitis (n = 81)		Children without rhinitis (n = 101)		P-value	OR _crude_ (95% CI)
	Number (%)	Infected (%)	Number (%)	Infected (%)		
Sex						
Male	44 (54.3)	6 (13.6)	57 (56.4)	2 (3.5)	0.77	1
Female	37 (45.7)	4 (10.8)	44 (54.3)	2 (4.5)		1.09 (0.61–1.96)
Age						
< 6	26 (32.1)	3 (11.5)	49 (48.5)	1 (2.0)	0.025	1
> 6	55 (67.9)	7 (12.7)	52 (51.5)	3 (5.8)		1.99 (1.09–3.66)
Residence						
Urban	58 (71.6)	7 (12.1)	52 (51.5)	1 (1.9)	0.006	2.38 (1.28–4.42)
Rural	23 (28.4)	3 (13.0)	49 (48.5)	3 (6.1)		1
Family income						
< 1500,000 T	56 (69.1)	7 (12.5)	73 (72.3)	3 (4.1)	0.64	1
≥ 1500,000 T	25 (30.9)	3 (12.0)	28 (27.7)	1 (3.6)		1.16 (0.61–2.21)
Dog contact						
No	76 (93.8)	8 (10.5)	88 (87.1)	4 (4.5)	0.13	1
Yes	5 (6.2)	2 (40.0)	13 (12.9)	0 (0.0)		0.45 (0.15–1.31)
Cat contact						
No	78 (96.3)	10 (12.8)	98 (97.0)	4 (4.1)	0.78	1
Yes	3 (3.7)	0 (0.0)	3 (3.0)	0 (0.0)		1.26 (0.25–6.40)
Frequent contact with the soil						
No	67 (82.7)	4 (6.0)	89 (88.1)	1 (1.1)	0.3	1
Yes	14 (17.3)	6 (42.9)	12 (11.9)	3 (25.0)		1.55 (0.67–3.57)
Eating unwashed vegetable						
No	77 (95.1)	6 (12.3)	95 (94.1)	4 (4.2)	0.77	1
Yes	4 (4.9)	4 (100.0)	6 (5.9)	0 (0.0)		0.82 (0.22–3.02)
Parents education						
Diploma and less	37 (45.7)	5 (13.5)	80 (79.2)	2 (2.5)	< 0.001	1
College and above	44 (54.3)	5 (11.4)	21 (20.8)	2 (9.5)		4.53 (2.37–8.68)
Mother’s occupation						
Employed	21 (25.9)	4 (19.0)	7 (6.9)	1 (14.3)	< 0.001	4.70 (1.88–11.73)
Housewife	60 (74.1)	6 (10.0	94 (93.1)	3 (3.2)		1
Father’s occupation						
Government employment and other	79 (97.5)	9 (11.4)	93 (92.1)	4 (4.3)	0.11	1
Agricultural activities	2 (2.5)	1 (50.0)	8 (7.9)	0 (0.0)		0.29 (0.06–1.43)
Water source						
Treated	67 (82.7)	6 (9.0)	89 (88.1)	2 (2.2)	0.3	1
Untreated	14 (17.3)	4 (28.6)	12 (11.9)	2 (16.7)		1.55 (0.67–3.56)
Family atopic history						
Yes	35 (43.2)	5 (14.3)	5 (5.0)	1 (20.0)	< 0.001	14.60 (5.37–39.7)
No	46 (56.8)	5 (10.9)	96 (95.0)	3 (3.1)		1
Parents smoking						
Yes	70 (86.4)	7 (10.0)	65 (64.4)	4 (4.6)		3.52 (1.65–7.50)
No	11 (13.6)	3 (27.3)	36 (35.6)	0 (0.0)	< 0.001	1

Statistically significant

**Fig. 1:**
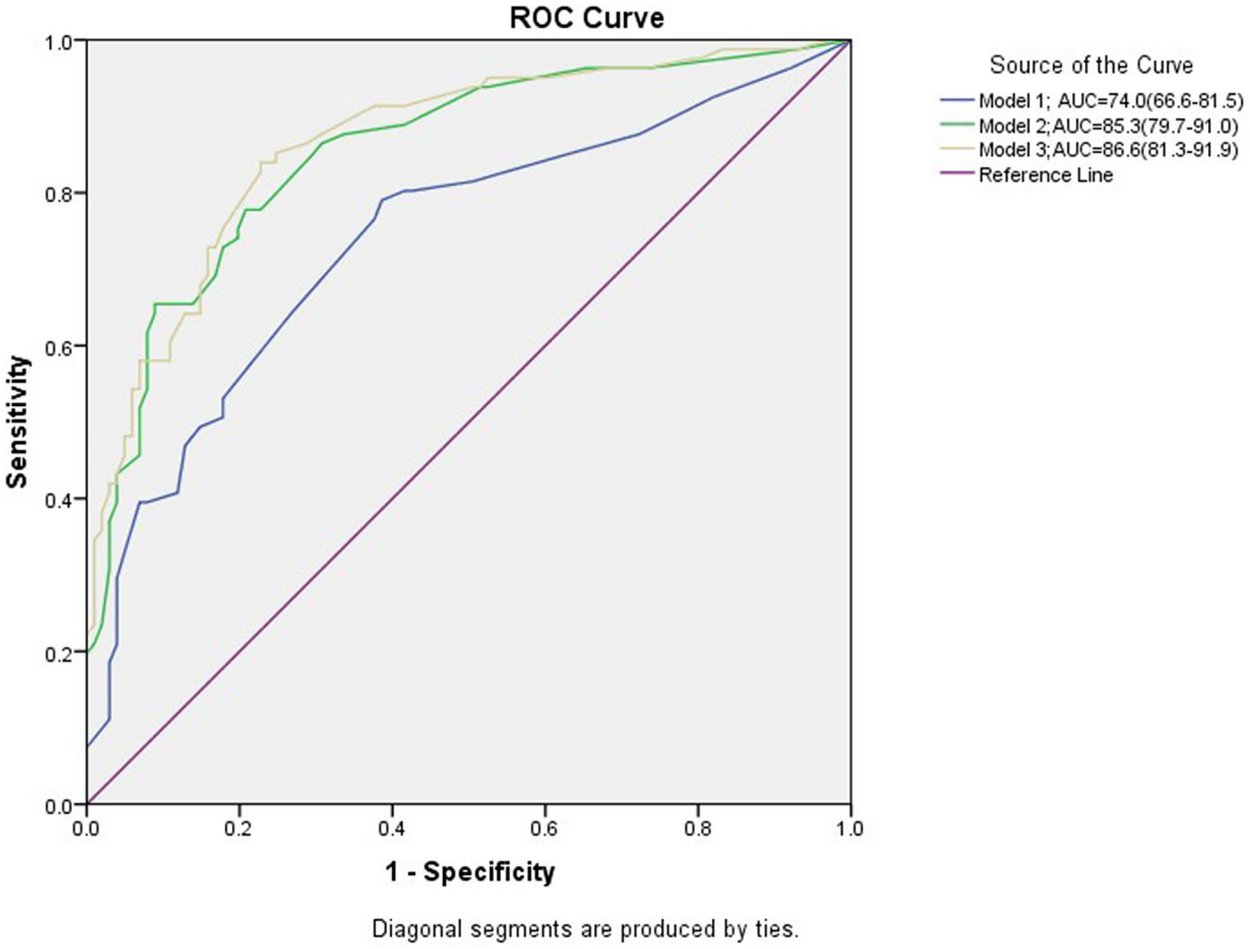
ROC curves for prediction models to discriminate rhinitis and *Ascaris* infection. Model 1 adjusted for age, sex, residence, parent’s education, mother occupation; model 2 adjusted for model 1 and family atopic history; model 3 adjusted for model 2 and parent’s smoking. Results show that variables in model 3 can explain 86.6% of the variance of the rhinitis. Therefore, this model is valid and considered as final model to multivariate analysis. The Hosmer and Lemeshow test also confirms the accuracy of the final model (P = 0.74). In the final modeling (model 3), sensitivity, specificity and accuracy for predicting rhinitis/healthy status were 71.6, 84.0 and 78.6%, respectively

Overall, *A. lumbricoides* seropositivity was higher in children with rhinitis (12.34%, 9.92–14.75%) than in healthy controls (3.96%, 95% CI 3.26–4.65%). Statistical analyses revealed that, however, seropositivity to *Ascaris* infection was significantly associated with childhood rhinitis in univariate analysis (OR, 3.42; 95% CI 1.03–11.3; *P* value = 0.035), but this association was no longer significant after adjustment for potential confounders (OR, 1.85; 95% CI 0.42–8.18; AUC = 86.6; 95% CI 81.3–91.9) (Table 2 and Fig. 1). In addition, there were 3/81 (3.7%, 95% CI 2.97–4.42) anti-*Toxocara* IgG seropositive children identified among the cases and 1/101 (0.99%, 95% CI 0.81–1.16%) among the rhinitis-free controls, indicating no significant association between *Toxocara* seropoitivity and rhinitis in both univariate analysis (OR, 3.84; 95% CI 0.39–37.7) and multivariate analysis after adjustment (OR, 0.8; 95% CI 0.04–15.44) (Table 2).

**Table 2:** Univariate and multivariate analyses for determination of association between rhinitis and Ascariasis and Toxocariasis

Variable	Children with rhinitis (n = 81)	Children without rhinitis (n = 101)	P-value	Univariate analyses	Multivariate analyses	Hosmer and Lemeshow Test for adjusted model
	Number (%)	Number (%)		ORs (95% CIs)	ORs (95% CIs)^a^	
Ascariasis						
Positive	10 (12.3)	4 (4.0)	0.035	3.42 (1.03–11.3)	1.85 (0.42–8.18)	0.68
Negative	71 (87.7)	97 (96.0)		1	1	
Toxocariasis						
Positive	3 (3.7)	1 (1.0)	0.22	3.84 (0.39–37.7)	0.80 (0.04–15.44)	0.74
Negative	78 (96.3)	100 (99.0)		1	1	

^a^Adjusted for Age, Sex, Residence, Parent’s education, Mother Occupation, Family atopic history, Parent’s smoking (Model 3 in Fig. 1)

## Discussion

To our present knowledge, the association of helminthes infection in development of allergic disorders is controversial. Besides that, epidemiological studies evaluating the role of *A. lumbricoides* and *Toxocara* infections in development of AR are very rare. Therefore, we designed and did a case–control study to further understanding this association. Our results demonstrated that, however, children with AR had a more exposure to *A. lumbricoides* (12.3% vs. 3.9%) and *Toxocara* spp. (3.7% vs. 0.99%) in comparison with healthy subjects, but a non-significant association was observed after adjustment for both of these parasitic infections.

In regard to *A. lumbricoides* infection, to our knowledge, this study is first to assess of a relationship between anti-*Ascaris* IgG and increased morbidity of AR. Two previous studies used specific IgE antibodies against *A. lumbricoides* to evaluate this association [21, 22]. In accordance with these studies, our univariate analysis showed a significant association between exposure to *A. lumbricoides* and development of AR in children, but when confounders were adjusted, in contrast with previous studies, this association was non-significant. With respect to *Toxocara* infection, previous studies showed contradictory results [19, 21, 30]. In accordance with our results, Arshi et al. [20] in Iran reported that although patients with AR had a higher seroprevalence rate of *Toxocara* infection in comparison with healthy controls, but difference was statistically non-significant. Mohammed Abdalla et al. in Egypt [21] and Yariktas et al. [17] in Turkey demonstrated a significant higher toxocariasis seropositivity rate in the patients with AR compared to controls. In contrast with above-mentioned studies, Manuel et al. [19] in Malaysia reported a significant higher seropositive rate of *Toxocara* infection in the controls as compared to allergic patients. More details for previous studies are presented in Table 3. The different results in studies could be due to difference in sample size and diagnostic criteria, difference in the genetic and immunological reactions, severity and susceptibility of recruited population to *Ascaris* infection from one to another setting.

**Table 3:** Previous case–control studies evaluating the role of Ascariasis and Toxocariasis in development of allergic rhinitis

Parasitic infection	Country/type of participants	Diagnostic antibody	Cases		Controls		Adjusted OR (95% CI)	P value
Author/references			Number	Infected, N (%)	Number	Infected, N (%)		
Ascariasis								
Zakzuk et al. [22]	Colombia/Rural population/all age	Sera/Specific IgE	89	50 (56.2)	146	58 (39.7)	2.04 (1.14–3.65)	0.02
Zakzuk et al. [22]	Colombia/Rural population/all age	Skin prick tests	90	22 (24.4)	153	19 (12.4)	2.21 (1.07–4.56)	0.03
Mohammed Abdalla et al. [21]	Egypt/children	Sera/Specific IgE	139	26 (18.7)	70	7 (10)	Not determined	0.01
Toxocariasis								
Yariktas et al. [30]	Turkey/all age	Sera/Specific IgG	64	18 (28.1)	61	7 (11.5)	Not determined	0.02
Yariktas et al. [30]	Turkey/all age	Sera/Specific IgE	64	11 (17.2)	61	2 (3.3)	Not determined	0.01
Manuel et al. [19]	Malaysia/all age	Sera/Specific IgG	85	14 (16.5)	85	32 (37.6)	Not determined	0.002 Negative association
Arashi et al. [20]	Iran/all age	Sera/Specific IgG	93	5 (5.4)	87	3 (3.4)	Not determined	0.39
Mohammed Abdalla et al. [21]	Egypt/children	Sera/Specific IgG	139	25 (18)	70	5 (7.1)	Not determined	0.01

The mechanism of pathogenesis, linking helminthes infections to allergic diseases is not clear, although it is assumed that immune response against these infections plays important role in development of allergic symptoms. It is hypothesized that these infections can influence allergic diseases by either stimulating or suppressing the allergic response, probably depending on the severity of the infection, host genetic susceptibility and the degree of exposure [16]. Immune responses raised against *Toxocara* and *Ascaris* infections are mediated by TH2 cell activation, which leads to a high concentration of IgE and eosinophil activation [16]. TH2 cells are associated with the high secretory level of IL-4, IL-13, and IL-5 cytokines. IL-4 and IL-13 stimulate polyclonal IgE production that binds to FcR on mucosal mast cells located in intestinal and alveoli tissue. IL-5 is also a potential factor for the development and activation of eosinophil [16, 31]. Besides, TH2 cells recruited in the site of allergic rhinitis also produce the same cytokine network. The pathological pathway involved in allergic rhinitis is probably started via mast cell activation and degranulation. Mast cells are activated through cross-linking of FcεRI, which occurs by binding of multivalent antigen to IgE [19, 32]. Moreover, some experimental studies have shown that infections with nematodes whose life cycle includes migration across different tissues (e.g. *Toxocara* spp. and *Ascaris suum*) could be associated with allergic disorders [33, 34]. On the other hand, because of inadequate innate immune response and parasite resistance to acquired immune response, most helminth infections are chronic. Chronic infections are associated with immune homeostasis. To modify the TH2 cell’s immune response, the basic level of IL-10 and TGF-β cytokines is produced by T regulatory (Treg) cells. Recent studies have shown that Treg cells in allergic diseases are dysfunctional, while Tregs of helminthic infections like *Toxocara* and *Ascaris* are more sufficient and functional [35]. So children who had exposure to *Toxocara* and *Ascaris* have more sufficient Tregs, which can compensate defects of allergic Treg cells. Hence the risk of AR in the face of helminth infection decreases with these justifications.

This study has some limitations. Low sample size is an important limitation, affection the significance of our results. We did not perform stool examination to determine whether there was evidence of chronic or active ascariasis. We also performed only a single ELISA on available sera without additional confirmative examinations, such as Western Blot or determination of IgE sensitization. Moreover, there is no supporting data on blood eosinophils or complete blood count (CBC) for our recruited population. Most of these limitations were related to our financial constraints.

## Conclusion

In conclusion, our results showed that, although non-significant, Iranian children with AR had more exposure to *A. lumbricoides* and *Toxocara* infection. These results suggest more experimental and epidemiological investigations to further elucidate this relationship.

## Data Availability

Data supporting the results of this article are included within the article. The raw datasets for this study are available from the corresponding author upon reasonable request.

## References

[1] Greiner AN, Hellings PW, Rotiroti G, Scadding GK (2011). Allergic rhinitis. Lancet.

[2] Dykewicz MS, Hamilos DL (2010). Rhinitis and sinusitis. J Allergy Clin Immunol..

[3] Meltzer E, Gross GN, Katial R, Storms W (2012). Allergic rhinitis substantially impacts patient quality of life: findings from the Nasal Allergy Survey Assessing Limitations. J Fam Pract.

[4] Blaiss MS, Hammerby E, Robinson S, Kennedy-Martin T, Buchs S (2018). The burden of allergic rhinitis and allergic rhinoconjunctivitis on adolescents: a literature review. Ann Allergy Asthma Immunol.

[5] Small P, Keith PK, Kim H (2018). Allergic rhinitis. Allergy Asthma. Clin Immunol..

[6] Van Mutius E, Martinez F, Adkinson NF, Yunginger JW, Busse WW, Bochner B, Holgate ST, Simons FER (2003). Natural history, development, and prevention of allergic disease in childhood. Middleton’s allergy: principles and practice.

[7] Bendtsen P, Grønbæk M, Kjær SK, Munk C, Linneberg A, Tolstrup JS (2008). Alcohol consumption and the risk of self-reported perennial and seasonal allergic rhinitis in young adult women in a population-based cohort study. Clin Exp Allergy.

[8] Aghamolaie S, Seyyedtabaei SJ, Behniafar H, Foroutan M, Saber V, Hanifehpur H (2018). Seroepidemiology, modifiable risk factors and clinical symptoms of *Toxocara* spp. infection in northern Iran. Trans R Soc Trop Med Hyg.

[9] Mohammadzadeh I, Rostami A, Darvish S, Mehravar S, Pournasrollah M, Javanian M (2019). Exposure to Ascaris lumbricoides infection and risk of childhood asthma in north of Iran. Infection.

[10] Rostami A, Riahi S, Holland C, Taghipour A, Khalili-Fomeshi M, Fakhri Y (2019). Seroprevalence estimates for toxocariasis in people worldwide: a systematic review and meta-analysis. PLoS Negl Trop Dis..

[11] Rostami A, Ma G, Wang T, Koehler AV, Hofmann A, Chang BC (2019). Human toxocariasis–A look at a neglected disease through an epidemiological ‘prism’. Infect Genet Evol..

[12] Fakhri Y, Gasser R, Rostami A, Fan C, Ghasemi S, Javanian M (2018). *Toxocara* eggs in public places worldwide—a systematic review and meta-analysis. Environ Pollut..

[13] Pullan RL, Smith JL, Jasrasaria R, Brooker SJ (2014). Global numbers of infection and disease burden of soil transmitted helminth infections in 2010. Parasite Vector..

[14] Aghaei S, Riahi SM, Rostami A, Mohammadzadeh I, Javanian M, Tohidi E (2018). *Toxocara* spp. infection and risk of childhood asthma: a systematic review and meta-analysis. Acta Trop..

[15] Mohammadzadeh I, Riahi SM, Saber V, Darvish S, Amrovani M, Arefkhah N (2018). The relationship between *Toxocara* species seropositivity and allergic skin disorders: a systematic review and meta-analysis. Trans R Soc Trop Med Hyg.

[16] Caraballo L, Acevedo N, Buendía E (2015). Human ascariasis increases the allergic response and allergic symptoms. Curr Trop Med Rep..

[17] Yariktas M, Demirci M, Aynali G, Kaya S, Doner F (2007). Relationship between *Toxocara* seropositivity and allergic rhinitis. Am J Rhinol Allergy..

[18] Leonardi-Bee J, Pritchard D, Britton J, Collaboration PiA (2006). Asthma and current intestinal parasite infection: systematic review and meta-analysis. Am J Respir Crit Care Med..

[19] Manuel AM, Kuljit S, Gopalakrishnan G, Suresh K, Balraj P (2012). The role of worm infestation in allergic rhinitis. Trop Biomed.

[20] Arshi S, Zandavar H, Oormazdi H, Akhlaghi L, Razmjou E, Hadighi R (2012). Study on the association of Toxocariasis with allergic rhinitis in individuals referred to Rasoul Akram Hospital, Tehran. Razi J Med Sci..

[21] Alam-eldin MA, Saad K, Abd-Elkader R, Yones D, Abdelmoghny A, Aboul-Khair MD (2019). Detection of Parasitic Infections in Children with Allergic Rhinitis Compared to Healthy Control in Upper Egypt. Iranian J Pediatr..

[22] Zakzuk J, Casadiego S, Mercado A, Alvis-Guzman N, Caraballo L (2018). *Ascaris lumbricoides* infection induces both, reduction and increase of asthma symptoms in a rural community. Acta Trop.

[23] Rostami A, Ebrahimi M, Mehravar S, Omrani VF, Fallahi S, Behniafar H (2016). Contamination of commonly consumed raw vegetables with soil transmitted helminth eggs in Mazandaran province, northern Iran. Int J Food Microbiol.

[24] Siyadatpanah A, Tabatabaei F, Emami ZA, Spotin A, Fallah OV, Assadi M et al. Parasitic contamination of raw vegetables in Amol, North of Iran. 2013.

[25] Yakhchali M, Rostami A, Esmaelzadeh M (2011). Diversity and seasonal distribution of ixodid ticks in the natural habitat of domestic ruminants in north and south of Iran. Revue Méd Vét..

[26] Zamanfar D, Ghaffari J, Behzadnia S, Yazdani-charati J, Tavakoli S (2016). The prevalence of allergic rhinitis, eczema and asthma in students of guidance schools in Mazandaran Province, Iran. Macedonian J Med Sci..

[27] Mohammadzadeh I, Ghafari J, Barari SKR, Tamadoni A, Esmaeili DM, Alizadeh NR (2008). The prevalence of asthma, allergic rhinitis and eczema in north of Iran: The international study of asthma and allergies in childhood (ISAAC). Iranian J Pediatr..

[28] Brożek JL, Bousquet J, Baena-Cagnani CE, Bonini S, Canonica GW, Casale TB (2010). Allergic Rhinitis and its Impact on Asthma (ARIA) guidelines: 2010 revision. J Allergy Clin Immunol..

[29] Nasiri S, Hedayati M, Riahi SM, Robati RM, Khazan M (2018). Elevated serum nitric oxide and hydrogen peroxide levels as potential valuable predictors of herpes zoster. Asian Pacific J Trop Med..

[30] Yariktas M, Demirci M, Aynali G, Kaya S, Doner F (2007). Relationship between *Toxocara* seropositivity and allergic rhinitis. Am J Rhinol..

[31] Ma G, Holland CV, Wang T, Hofmann A, Fan C-K, Maizels RM (2018). Human toxocariasis. Lancet Infect Dis..

[32] Sereda MJ, Hartmann S, Lucius R (2008). Helminths and allergy: the example of tropomyosin. Trends Parasitol..

[33] Pinelli E, Withagen C, Fonville M, Verlaan A, Dormans J, Van Loveren H (2005). Persistent airway hyper-responsiveness and inflammation in Toxocara canis-infected BALB/c mice. Clin Exp Allergy.

[34] Pritchard D, Eady R, Harper S, Jackson D, Orr T, Richards I (1983). Laboratory infection of primates with *Ascaris suum* to provide a model of allergic bronchoconstriction. Clin Exp Immunol.

[35] Sitcharungsi R, Sirivichayakul C (2013). Allergic diseases and helminth infections. Pathog Global Health..

